# Exposure to Sub-Lethal Doses of Permethrin Is Associated with Neurotoxicity: Changes in Bioenergetics, Redox Markers, Neuroinflammation and Morphology

**DOI:** 10.3390/toxics9120337

**Published:** 2021-12-06

**Authors:** Teresita Guadalupe López-Aceves, Elvia Coballase-Urrutia, Francisco Estrada-Rojo, América Vanoye-Carlo, Liliana Carmona-Aparicio, María Eugenia Hernández, José Pedraza-Chaverri, Luz Navarro, Omar E. Aparicio-Trejo, Armando Pérez-Torres, Omar N. Medina-Campos, Daniel Martínez-Fong, Vicente Sánchez-Valle, Noemi Cárdenas-Rodríguez, Leticia Granados-Rojas, Evelyn Pulido-Camarillo, Verónica Rodríguez-Mata, Claudia del R. León-Sicairos

**Affiliations:** 1 Regional Graduate Program in Biotechnology, Faculty of Biological Chemical Sciences, Autonomous University of Sinaloa, Culiacán 80000, Mexico; tgla19@hotmail.com (T.G.L.-A.); claudialeonsicairos@uas.edu.mx (C.d.R.L.-S.); 2 Laboratory of Neuroscience, National Institute of Pediatrics, Mexico City 04530, Mexico; america_vc@yahoo.com.mx (A.V.-C.); c_apariccio@yahoo.com.mx (L.C.-A.); noemicr2001@yahoo.com.mx (N.C.-R.); lgranados_2000@yahoo.com.mx (L.G.-R.); 3 Department of Physiology, Faculty of Medicine, National Autonomous University of Mexico, Mexico City 04510, Mexico; fesro@hotmail.com (F.E.-R.); navarroluz2002@yahoo.com.mx (L.N.); 4 Subdirection of Clinical Research, National Institute of Psychiatry, Mexico City 14370, Mexico; drosoph2001@yahoo.com.mx; 5 Department of Biology, Faculty of Chemistry, National Autonomous University of Mexico, Mexico City 04150, Mexico; pedraza@unam.mx (J.P.-C.); emilianoaparicio91@gmail.com (O.E.A.-T.); omarnoelmedina@gmail.com (O.N.M.-C.); 6 Department of Cell and Tissue Biology, Faculty of Medicine, National Autonomous University of Mexico, Mexico City 04510, Mexico; armandop@unam.mx (A.P.-T.); elyn_21@live.com.mx (E.P.-C.); verohistologabct@hotmail.com (V.R.-M.); 7 Department of Physiology, Biophysics and Neurosciences, Center for Research and Advanced Studies, Mexico City 07360, Mexico; dmartine@fisio.cinvestav.mx; 8 Neuroplasticity and Neurodegeneration Laboratory, Department of Pharmacology, Center for Research and Advanced Studies, Mexico City 07360, Mexico; vicente2779@hotmail.com

**Keywords:** permethrin, neurotoxicity, bioenergetics, neuroinflammation

## Abstract

Permethrin (PERM) is a member of the class I family of synthetic pyrethroids. Human use has shown that it affects different systems, with wide health dysfunctions. Our aim was to determine bioenergetics, neuroinflammation and morphology changes, as redox markers after subacute exposure to PERM in rats. We used MDA determination, protein carbonyl assay, mitochondrial O_2_ consumption, expression of pro-inflammatory cytokines and a deep histopathological analysis of the hippocampus. PERM (150 mg/kg and 300 mg/kg body weight/day, o.v.) increased lipoperoxidation and carbonylated proteins in a dose-dependent manner in the brain regions. The activities of antioxidant enzymes glutathione peroxidase, reductase, S-transferase, catalase, and superoxide dismutase showed an increase in all the different brain areas, with dose-dependent effects in the cerebellum. Cytokine profiles (IL-1β, IL-6 and TNF-α) increased in a dose-dependent manner in different brain tissues. Exposure to 150 mg/kg of permethrin induced degenerated and/or dead neurons in the rat hippocampus and induced mitochondrial uncoupling and reduction of oxidative phosphorylation and significantly decreased the respiratory parameters state 3-associated respiration in complex I and II. PERM exposure at low doses induces reactive oxygen species production and imbalance in the enzymatic antioxidant system, increases gene expression of pro-inflammatory interleukins, and could lead to cell damage mediated by mitochondrial functional impairment.

## 1. Introduction

The potential risks of exposure to different pesticides have been the subject of multiple investigations. Different studies reported their effects on physiology, which can lead to the development of chronic degenerative diseases with epidemiological impact [1,2,3]. The residues of these compounds have been reported in food, water, sea life, birds, and biological substrates such as different tissues and breast milk [4,5,6,7,8].

Pyrethroid pesticides are synthetic derivatives of natural pyrethrins obtained from the chrysanthemum flower *Chrysanthemum cinerariefolium*. Pyrethroids include 42 compounds divided into two types and originally were considered safe for humans and other animals [9]. These compounds have become first-line insecticides for the control of domestic pests. They often replace home and agricultural use of certain restricted or banned insecticides, such as organophosphates and organochlorine [9,10,11,12]. Due to the constant use of pyrethroids in agricultural activities and in homes, they constitute a danger due to their accumulation in solid food [13].

The use of type I pyrethroids has been associated with tremors of the whole body, aggressive behavior, hypersensitivity and ataxia [9,11,12,13,14,15]. Their mechanism of action is related to changes in sodium channel conformation during their opening and closing in neuronal membranes [14,15]. Conversely, type II pyrethroids can cause choreoathetosis-salivation syndrome and motor dysfunction possibly due to effects on chloride channels, including γ-aminobutyric acid (GABA) receptors [14].

Permethrin (PERM) is a member of the family of synthetic class I pyrethroids. It is widely used in agriculture and residential homes, since it is found in household sprays, aerosols, insect repellents, pet shampoos and lotions. It is also used directly in humans, as a treatment to eradicate dermal parasites such as scabies and pediculosis (lice) [16,17,18,19,20]. PERM has been shown to affect the reproductive, skeletal, cardiovascular, immune and neuronal systems by generating cardiotoxicity, endocrine dysfunction, hepatotoxicity and cytotoxicity [16,21,22,23,24,25].

PERM may enter the body by skin contact, inhalation or ingestion (water/food) and due to its lipophilicity, can pass through the blood–brain barrier at concentrations that might be neurotoxic. It has been reported that PERM acts on glia and neurons, decreases neurogenesis and causes a partial loss of neurons and mild inflammation. It increases proinflammatory cytokines, mainly Tumor Necrosis Factor-alpha (TNF-α), and contributes to neuroinflammation underlying several neurodegenerative diseases. Using in vitro and animal models, studies of pyrethroid effects showed that it activates microglia and the release of proinflammatory cytokines, as well as chronic neuroinflammation [26]. In Gulf War veterans, it has been reported that pyrethroids alter the blood–brain barrier, allowing the entrance of pro-inflammatory cytokines into the circulating blood, where they can act on peripheral immune cells and induce immune modulation [22,27,28,29,30].

The brain is especially vulnerable to mitochondrial dysfunction because it requires more energy than any other organ in the body. In this process, reactive oxygen species (ROS) are produced by the electron transport system elements, particularly by complex I and, to a lesser extent, by complex III. Recent studies have shown that exposure to different permethrin concentrations can alter the function of mitochondria, with the consequent increase in oxidative stress and inflammatory processes [27,28,30]. PERM can adversely affect cognitive and mood function either directly or indirectly via reduced hippocampal neurogenesis [27,28,29,30,31]. In the present study, we evaluated neurotoxicity induced by subacute exposure to PERM in sublethal doses and its effects on the immune systems, bioenergetic redox and morphology of the brain using an animal model.

## 2. Materials and Methods

All the experimental procedures followed the guidelines of the Official Mexican Norm for the use and care of laboratory animals (NOM-062-ZOO-1999), as well as for the disposal of biological residues (NOM-087-ECOL-1995). Experimental procedures are part of project 060/2018, approved by the Institutional Committees of Research and of Laboratory Animal Use and Care. Institutional Committee of Research is registered at the NIH Office for Human Research Protection (http://ohrp.cit.nih.gov/search (accessed on 31 July 2020) with number IRB00008064 on 16 January 2019.

### 2.1. Drugs

All reagents and chemicals were purchased from Sigma-Aldrich (St. Louis, MO, USA). Sodium pentobarbital was obtained from Pisabental^®^, PiSA Agropecuaria (Guadalajara, Jalisco, Mexico). All analytical reagents and stains were purchased from J.T. Baker (Xalostoc, Estado de México, Mexico). All other chemicals used in this study were of reagent grade and were commercially available.

### 2.2. Experimental Groups

Male Wistar rats weighing 150–180 g (5–6 weeks old) were used. The animal-housing room was maintained under constant conditions of temperature (21 ± 1 °C), relative humidity (50–60%) and lighting (12-h light/dark cycle). Filtered air (5 mm particles) was exchanged 18 times/h. Animals were provided with a standard commercial rat chow diet (Harlan Teklad Global diet 2018S sterilized, Harland Teklad, Madison, WI, USA), and reverse osmosis filtered water was used. Rats were divided into four groups: Group (1) Sham (manipulated animals, no administration); Group (2) Animals with vehicle treatment (corn oil 1 mL/kg/weight); Group (3) Animals with 150 mg/kg, orally/day of PERM + vehicle; Group (4) Animals with 300 mg/kg, orally/day PERM + vehicle and treated every 24 h for 14 days. Forty-eight hours after the last dose of permethrin, all animals were anesthetized with pentobarbital (0.6 μL/kg, i.p.).

The brains (cerebellum, cortex, cerebral hemispheres and medulla) were immediately excised and stored at −70 °C until analysis of antioxidant enzymes, protein carbonylation (PC) and lipid peroxidation, as well as pro-inflammatory cytokine expression by RT-PCR (*n* = 6 per group). In order to evaluate effects of PERM on bioenergetics, brain mitochondria were isolated and O_2_ consumption was measured.

### 2.3. Permethrin Oral Exposure

PERM was dissolved in corn oil and administered orally (*n* = 6) for 15 days, at daily doses of 150 mg/kg body weight per day (1/10 of LD50) and 300 mg/kg body weight/day (1/5 of LD50) [32]. The vehicle group received corn oil (1 mL/kg, *n* = 12) for 15 days. The compounds were administered orally using an intragastric tube in the morning, and rats were not fasted overnight.

### 2.4. Tissue Homogenization

Tissues were homogenized in phosphate buffer (0.1 M, pH 7.0) with 0.1% Triton X-100 using a Brinkmann Polytron model PT 2000 (Westbury, NY, USA) and centrifuged at 19,000X *g* for 10 min. The supernatant was used to quantify total protein concentration (Lowry method), evaluate lipoperoxidation (MDA), protein carbonyl content and the activity of antioxidant enzymes, such as superoxide dismutase (SOD), catalase (CAT), glutathione peroxidase (GPx), glutathione reductase (GR), and glutathione S-transferase (GST).

### 2.5. Total Protein Quantitation by the Lowry Method

Samples subjected to this colorimetric reaction were read in triplicate on a spectrophotometer (BioTek; Synergy HT) at 660 nm. Protein quantification was performed using an 8-point standard curve of bovine serum albumin (BSA), which was used as a reference [33].

### 2.6. Oxidative Stress Marker Detection

Determination of MDA levels was performed as described by Coballase-Urrutia et al. (2010 and 2013) [34,35]. The activity of the antioxidant enzymes GR, SOD, CAT and GPx (all in U/mg protein) and GST (µmol CDNB conjugate formed/min/mg protein) was measured.

### 2.7. Protein Carbonyl Assay

Protein carbonyl content was analyzed using a PC ELISA kit (Enzo Life Sciences, Plymouth Meeting, PA, USA). Protein carbonyl levels were expressed as nM/mg of protein [36].

### 2.8. Mitochondrial Isolation

After animal sacrifice, the hippocampus was cooled to 4 °C by immersion in mitochondrial isolation buffer (225 mM D-mannitol, 75 mM sucrose, 1 mM, Ethylenediamine tetra acetic acid (EDTA), 5 mM, 4-(2-hydroxyethyl)-1-piperazineethanesulfonic acid (HEPES), 0.1% BSA, pH = 7.4) and cut into small pieces. The tissues were homogenized in a glass Potter–Elvehjem with a TeflonVR pestle using the same buffer, and mitochondria were obtained by differential centrifugation [37]. Briefly, homogenates were centrifuged at 2000× *g* for 5 min, and the supernatants were centrifuged at 12,000× *g* for 15 min, after which a wash was performed with BSA-free mitochondrial isolation buffer [38]. The final pellet was resuspended in 80 µL of BSA-free mitochondrial isolation buffer and total mitochondrial proteins were estimated by the Lowry method [33].

### 2.9. Mitochondrial O_2_ Consumption

Mitochondrial O_2_ consumption was evaluated using high-resolution respirometry (oxygraph O2k, OROBOROS, Innsbruck, Austria) at 37 °C. Isolated mitochondria (300 µg of total protein) were loaded into a chamber with 2 mL of MiR05 respiration buffer: 0.5 mM EGTA, 3 mM MgCl_2_, 60 mM K-lactobionate, 20 mM taurine, 10 mM KH_2_PO_4_, 20 mM HEPES, 110 mM sucrose and 1 g/L essentially fatty-acid free BSA. Electron transport was started by addition of complex I-linked substrates (5 mM sodium pyruvate and 2 mM malate) or a complex II-linked substrate (10 mM succinate) plus a complex I inhibitor (0.5 µM rotenone). State 3 (S3) was stimulated by the addition of 2 mM ADP and 2.5 μM oligomycin induced state 4 (S4o). All parameters were corrected by residual respiration (ROX), which was obtained by addition of 0.5 µM rotenone plus 2.5 µM antimycin A. The respiratory control index was defined as the S3/S4o ratio, and OXPHOS-associated respiration (P) was defined as S3-S4o [37,38]. All reported values were normalized by total protein content estimated by the Lowry method [33].

### 2.10. RT-PCR Expression for Pro-Inflammatory Cytokines

Total RNA was isolated from 50–100 mg of frozen cerebellum, cerebral hemispheres, cortex and medulla by homogenization in Trizol TM (Invitrogen Corporation, Carlsbad, CA, USA) followed by chloroform-isopropanol extraction. RNA concentration was quantified using a NanoDrop (ND-1000 Spectrophotometer). RNA samples were treated with 1 U RNase-free DNase I (Invitrogen Life Technologies, Carlsbad, CA, USA) per 1 µg of RNA. Total RNA was used for the generation of cDNA by retro-transcription (SuperScript IIITM First-Strand Synthesis System for RT-PCR, InvitrogenTM) using 3 µg of total RNA and 0.1 mg oligo dT (Invitrogen Corporation, Carlsbad, CA, USA). The design of oligonucleotides was performed with the IDT program SciTools Primer Quest YE (Integrated DNA Technologies, Inc. © Copyright 2012). The reverse-transcribed product (cDNA) was amplified by PCR in a final reaction volume of 25 µL containing 2.5 µL of 10× buffer, 0.75 µL of MgCl2 50 mM, 0.5 µL of the forward oligonucleotide and 0.5 µL of the reverse oligonucleotide, 1.5 µL of cDNA, 0.5 µL of deoxyribonucleotide and 0.2 µL of Taq-polymerase (5 U/µL). The PCR was performed using a thermocycler (Veriti^®^ Thermal Cycler de Applied Biosystems) with an initial denaturation (94 °C, 5 min) followed by 30 cycles of denaturation (94 °C, 30 s), annealing (to align using corresponding Tm for 30 s), extension (72 °C, 20 s) and an additional denaturation (72 °C, 1 min).

The PCR products were separated on a 2% agarose gel and stained with ethidium bromide. Photographs of the gels were taken with a Kodak EDAS 290 camera (Kodak Co.; Rochester, NY, USA). PCR data analysis was carried out by normalization to beta-actin as a control gene in triplicate, and the expression levels were calculated by normalizing relative expression values to the mean of the untreated control. The sequences for each gene were searched in the National Center for Biotechnology Information (NCBI) database (data shown in Table 1).

### 2.11. Hippocampal Histopathological Analysis

Whole-body perfusion was performed in appropriately anesthetized rats (pentobarbital overdose) to fix the tissue. The pentobarbital overdose was administered in the experimental rats with 50 mg/kg i.p. of sodium pentobarbital (Pisabental^®^). A gravity-fed perfusion apparatus was used with two bottles, one containing physiological solution and the other containing fixative solution; these were placed about 130 cm above the animal being perfused [39]. Blood was removed from the circulatory system with the physiological solution at room temperature (RT). Heparin was perfused in the left ventricle of the heart, with medical infusion equipment. A cut made in the right atrium allowing the exit of blood and saline solution for 20 min. Paraformaldehyde (4% *v*/*v*) in phosphate buffer (0.1 M, pH 7.2) at RT was perfused for 20 min. The rats were decapitated, and their brains were removed from the skull and immersed in the same fixative for 24 h at RT. After an overnight wash in tap water, the brains were manually sectioned with a blade, coronally at the bregma −2.12 mm stereotaxic coordinate [40]. The specimens were then processed by paraffin embedding to obtain tissue sections (4 µm thick) until the bregma −3.03 mm coordinate. Sections were stained with hematoxylin & eosin (HE) using the Klüver-Barrera method [41]. HE-stained sections were evaluated to detect necrotic neurons based on nuclear changes indicative of cell death such as pyknosis, karyolysis and karyorrhexis, attributable to the nonspecific breakdown of DNA. Furthermore, an increase in cytoplasmic eosinophilia was a histological manifestation of necrosis [42]. These histopathological findings were confirmed with the Klüver-Barrera method. Hippocampus of 6 animals per treatment were analyzed as follows. Three tissue sections (4 μm) of medial hippocampus (Bregma 3.3) containing Cornu Ammonis CA1, CA2, CA3 fields, as well as the superior and inferior blade and hinge of the dentate gyrus (DG), were analyzed. HE-stained sections were evaluated to detect necrotic neurons based on nuclear changes indicative of cell death such as pyknosis, karyolysis and karyorrhexis, attributable to the nonspecific breakdown of DNA. Furthermore, an increase in cytoplasmic eosinophilia is a histological manifestation of necrosis [42]. These histopathological findings were confirmed with Klüver-Barrera method.

### 2.12. Statistical Analysis

The statistical analysis was performed using GraphPad Prism 6.0 for Windows (GraphPad Software, San Diego, CA, USA). Results were expressed as mean ± standard deviations (mean ± SDM). Multiple comparisons were analyzed using one-way ANOVA followed by Bonferroni’s multiple comparisons post-hoc tests. To evaluate mitochondrial O_2_ consumption, a one-way ANOVA was performed followed by a Tukey pos-hoc test. Values of *p* < 0.05 were considered statistically significant.

## 3. Results

### 3.1. Permethrin Exposure Induces Lipoperoxidation and Carbonylation

**Figure 1:**
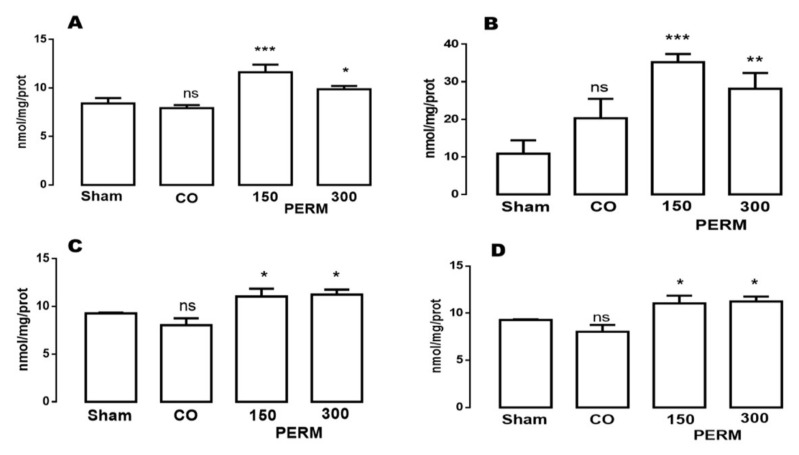
Effect of permethrin on protein carbonyl content in different tissues of the brain: (**A**) cerebellum; (**B**) cerebral cortex; (**C**) cerebral hemispheres; (**D**) medulla; at dose of 150 (1/10 of LD50) and 300 mg/kg body weight/day (1/5 of LD50). Corn oil (CO) was administered as vehicle (1 mL/kg/day) for 15 days. The results were analyzed by ANOVAs; Bonferroni’s multiple comparisons test was used to compare the outcomes between the experimental, vehicle and sham groups. Mean ± standard deviations (*n* = 6 per group). Without statistically different is showed as ns. *** *p* < 0.0001 vs. sham group; ** *p* < 0.001 vs. sham group, * *p* < 0.01 vs. sham group; ns (not significant). CO: Corn oil, PERM: Permethrin.

Lipoperoxidation and carbonylation were evaluated in the different brain areas of sham, vehicle (corn oil) and treated (150 or 300 mg/kg body weight permethrin) rats, after 15 days of treatment. The areas analyzed were cerebellum, cerebral cortex, cerebral hemispheres, and medulla (Table 2 and Figure 1). MDA values detected in the brain tissue of sham and vehicle groups were in the physiological range (baseline). PERM oral administration, at dose of 150 mg/kg body weight and 300 mg/kg body weight/day, increased lipoperoxidation in a dose-dependent manner in the brain regions analyzed (Table 2). The increase in lipoperoxidation after 150 mg/kg of permethrin exposure was 33.41% and 22.89% for the cerebellum and cortex, respectively, whereas for the cerebral hemispheres the increase was 69.88% and 59.73%, respectively, in the medulla when compared to vehicle and sham conditions.

The oxidative effect of PERM on the different brain areas was more evident using the 300 mg/kg dose; an increase of 44.17% was observed in the cerebellum, 40.61% in the cortex, 72.16% in the medulla and almost 100% in cerebral hemispheres.

In the four brain regions analyzed (cerebellum, cerebral cortex, cerebral hemispheres, and medulla) of the sham group, PC levels were low and considered as physiological values (Figure 1). On the other hand, carbonylated proteins detected in brain areas of animals treated with PERM showed an important increase compared to basal conditions. PERM treatment triggered a 37.47 and 20.80% increase in carbonylated proteins in the cerebellum with 150 mg/kg and 300 mg/kg, respectively (F(3,8) = 29.61, * *p* < 0.0001). For the cerebral cortex, the increase reached almost 100% with the 150 mg/kg dose and 84.6% with 300 mg/kg (F(3,8) = 21.06, * *p* < 0.0001). In the cerebral hemispheres, increases of 18.98% and 21.12% were observed (F(3,8) = 19.26, * *p* < 0.0001), and in the medulla, the increase was 28.0% and 50.56% with 150 mg/kg and 300 mg/kg, respectively (F(3,8) = 8.20,* *p* < 0.001, Figure 1).

### 3.2. Antioxidant Marker Determination

To deepen understanding of the mechanism of PERM-induced damage, GPx, GR, CAT, SOD and GST activities were evaluated in different brain regions, in consideration that these enzymes are the brain natural antioxidant system. PERM administration in different concentrations increased the activity of almost all the antioxidant enzymes explored in the different regions of the brain (cerebellum, cerebral cortex, cerebral hemispheres and medulla). The levels of activity of antioxidant enzymes in the brain regions of sham and corn oil treatment animals were similar and considered as physiological values (Table 3).

The activity of GPx increased after administration of both PERM doses (150 and 300 mg/kg). The percentages of increase when compared to sham values were: in the cerebellum, 36.84% and 52.63%, respectively (F(3,8) = 10,26, *p* < 0.001); prefrontal cortex, 41.66% and 33.33%, respectively (F(3,8) = 7.42, *p* < 0.01); cerebral hemispheres, 24.0% and 33.0%, respectively (F(3,8) = 7.96, *p* < 0.001); and in the medulla, a lower increase of 13.63% with both doses was detected (F(3,8) = 3.26, *p* < 0.001).

The activity of GR also increased with both PERM doses used (150 and 300 mg/kg) but was significant only in the cerebellum (46.15% and 61.53%, respectively; F(3,8) = 7.24, *p* < 0.01) and medulla (22.58% and 29.03%, respectively; F(3,8) = 9.81, *p* < 0.01). In the prefrontal cortex and cerebral hemispheres, the changes in activity were not significant.

For CAT activity, a dose-dependent effect was observed in the cerebellum and medulla, where the increase in enzyme activity was 56.85 and 24.27% for 150 mg/kg PERM (F(3,8) = 7.36, *p* < 0.01) and 73.93% and 29.18% for 300 mg/kg (F(3,8) = 8.05, *p* < 0.001). On the other hand, the higher PERM dose (300 mg/kg) induced a lower increase in CAT activity compared to the lower dose (150 mg/kg) in the prefrontal cortex (33.18% and 23.40%, respectively; F(3,8) = 11.38, *p* < 0.01) and in the cerebral hemispheres (49.17 and 48.6%, respectively; F(3,8) =7.06, *p* < 0.01).

The changes in SOD activity were the highest for both doses of PERM. In cerebellum the increases in SOD activity were 83.88% for 150 mg/kg and 85.78% for 300 mg/kg (F(3,8) = 15.86, *p* < 0.001). In the prefrontal cortex, SOD activity was 56.30% and 45.37% higher when compared to basal conditions (F(3,8) = 11.38, *p* < 0.001). For the cerebral hemispheres, the increase was lower than in other brain areas (26.74% and 30.23%; F(3,8) = 8.44, *p* < 0.001), and in the medulla, it was 77.77% and 100%.

GST activity in the cerebellum showed an increase of 50.0 and 88.0% for 150 and 300 mg/kg of PERM, respectively, compared to sham values (F(3,8) = 7.65, *p* < 0.0001). In the prefrontal cortex, the increase was 52.0% and 57.0%, respectively (F(3,8) = 9.07, *p* < 0.0001). In the cerebral hemispheres, GST activity was 45% higher with both doses, while in the medulla there were no significant changes.

### 3.3. Permethrin Stimulates Cytokine Gene Expression

**Figure 2:**
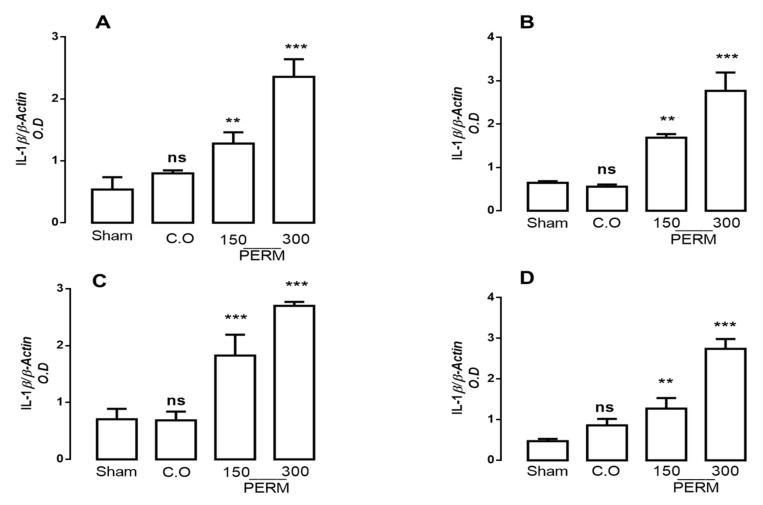
mRNA expression for IL-1β in the cerebellum (**A**), cerebral cortex (**B**), cerebral hemispheres (**C**) and medulla (**D**) in rats treated with corn oil (control group), permethrin 150 mg/kg or 300 mg/kg, and rats without treatment (sham group). Values are mean ± SD (*n* = 4 for each group). The results were analyzed with ANOVAs followed by Bonferroni’s multiple comparisons tests to compare permethrin (150 or 300 mg/kg) groups versus the control group (sham). Data from 6 independent experiments run in quadruplicate. Without statistically different is showed as ns. ** *p* < 0.001 vs. sham group; and *** *p* < 0.0001 vs. sham group; CO (Corn oil); PERM (permethrin).

**Figure 3:**
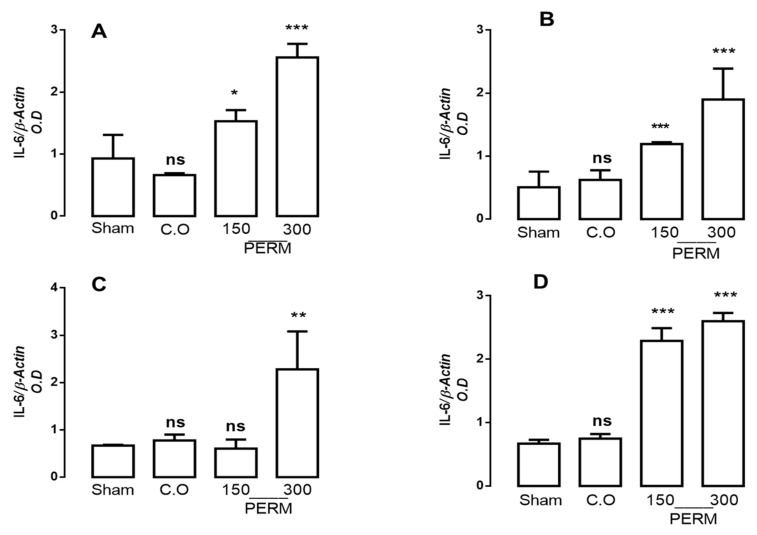
mRNA expression for IL-6 in the cerebellum (**A**), cerebral cortex (**B**), cerebral hemispheres (**C**) and medulla (**D**) in rats treated with corn oil (control group), permethrin 150 mg/kg or 300 mg/kg and rats without treatment (sham group). Values are mean ± SD (*n* = 4 for each group). The results were analyzed with ANOVAs followed by Bonferroni’s multiple comparisons tests to compare permethrin (150 or 300 mg/kg) groups versus the control group (sham). Data from 6 independent experiments run in quadruplicate. Without statistically different is showed as ns. * *p* < 0.05 vs. sham group, ** *p* < 0.001 vs. sham group; and *** *p* < 0.0001 vs. sham group; CO (Corn oil); PERM (permethrin).

**Figure 4:**
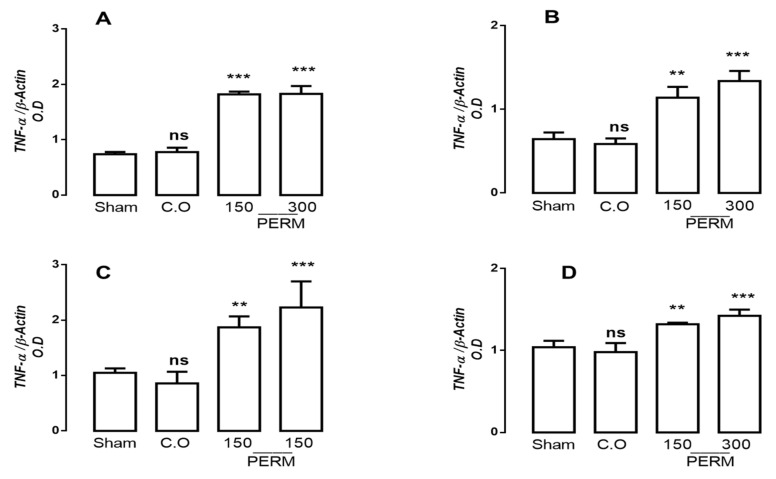
mRNA expression for TNFα in the cerebellum (**A**), cerebral cortex (**B**), cerebral hemispheres (**C**) and medulla (**D**) in rats treated with corn oil (control group), permethrin 150 mg/kg or 300 mg/kg and rats without treatment (sham group). Values are mean ± SD (*n* = 4 for each group). The results were analyzed with ANOVAs followed by Bonferroni’s post-hoc tests to compare permethrin (150 or 300 mg/kg) groups versus the control group (corn oil). Mean ± SD of 6 independent experiment run in quadruplicate. Without statistically different is showed as ns. ** *p* < 0.001 vs. sham group; and *** *p* < 0.0001 vs. sham group; CO (Corn oil); PERM (permethrin).

PERM exposure is related to the development of degenerative diseases, and pro-inflammatory cytokines have been found to be involved in their physiopathology. Thus, we assessed if PERM treatment changed cytokine profiles in the different brain tissues studied (cerebellum, cerebral cortex, cerebral hemispheres and medulla). IL-1β, IL-6 and TNF-α gene expression were analyzed by RT-PCR using the beta-actin gene for normalization (Figure 2, Figure 3 and Figure 4).

IL-1β gene expression increased significantly after PERM exposure at both concentrations in all brain regions analyzed (cerebellum, cerebral cortex, cerebral hemispheres and medulla) compared to control values (sham and corn oil, which were not significantly different; Figure 2). The biggest changes in IL-1β were found in the cerebellum and medulla, with an increase of 192.62% and 217.79%, respectively, after 150 mg/kg PERM (F(3,8) = 49.79, *p* < 0.0001) and 485.25% and 480.5%, respectively, for 300 mg/kg PERM (F(3,8) = 77.19, *p* < 0.0001). For the cerebral cortex and cerebral hemispheres, levels of IL-1β expression reached 160.06% and 158.52%, respectively, compared to control values with 150 mg/kg PERM (F(3,8) = 67.53, *p* < 0.0001) and 158.52% and 283.52%, respectively, with the higher dose (F(3,8) = 57.87, *p* < 0.0001).

IL-6 expression levels showed an important increase compared to basal conditions in the cerebellum, cerebral cortex and medulla after 150 mg/kg PERM (62.93, 135.64 and 244.28% respectively), while the increase after 300 mg/kg PERM was significant in all areas (166.4–287.28%; Figure 3; cerebellum: F(3,8) = 37.69, *p* < 0.001; cerebral cortex: F(3,8) = 14.94, *p* < 0.001; cerebral hemispheres: F(3,8) = 11.14, *p* < 0.001; medulla: F(3,8) = 186.1, *p* < 0.0001). No significant differences were found between the sham and CO groups.

TNF-α expression showed significant increases in all regions after PERM exposure at both concentrations. The greatest change was observed in cerebellum, where the increase in gene expression was 146.27 and 147.63% after 150 and 300 mg/kg permethrin, respectively (Figure 4A; F(3,8) = 150.6, *p* < 0.001). In the other areas, slight changes were observed: cerebral cortex (77.01% and 108.0%, respectively; F(3,8) = 39.15, *p* < 0.0001), cerebral hemispheres (78.09% and 112.38%, respectively; F(3,8) = 16.47, *p* < 0.0001) and medulla (15.78% and 24.56%, respectively; F(3,8) = 22.63, *p* < 0.001). No significant differences were found between the sham and control groups.

### 3.4. Histopathological Analysis

**Figure 5:**
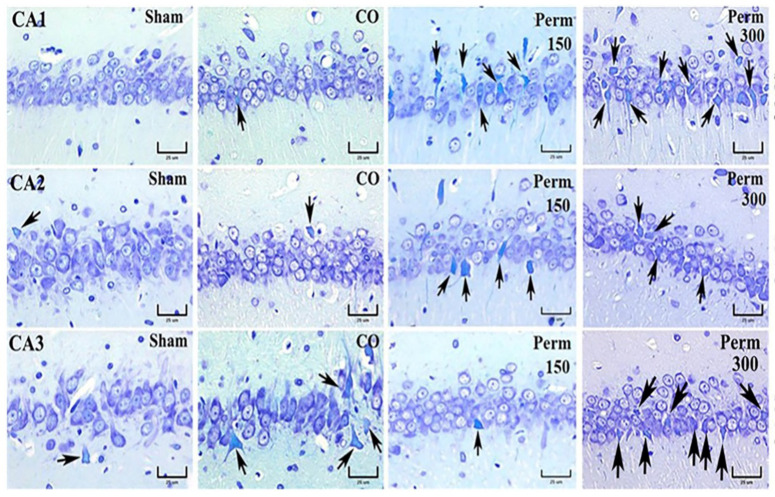
Photomicrographs of the hippocampal CA1, CA2 and CA3 of sham rats, rats treated with corn oil (CO) and rats treated with 150 and 300 mg/kg of permethrin. Arrows indicate neurons with histopathological features of cell degeneration or death. Klüver-Barrera stain.

**Figure 6:**
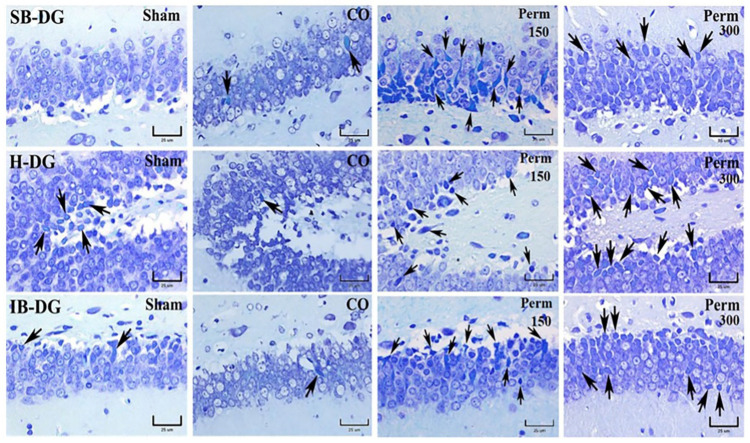
Photomicrographs of the superior lamina (SB-DG), hinge region (H-DG) and the inferior lamina (IB-DG) of the hippocampal dentate gyrus of rats with or without treatment. Arrows indicate neurons with histopathological features of cell degeneration or death by Klüver-Barrera stain.

The previous data showed an effect on the brain, even using the low dose of permethrin (150 mg/kg). We explored brain damage by histopathological techniques using the lower dose employed in this study. The degenerated and/or dead neurons, identified by cell body contraction, pyknotic cones as well as nuclei with karyorrhexis and karyolysis increased in different areas of the rat hippocampus (CA1, CA2, CA3 and DG) after exposure to permethrin (150 mg/kg) (Figure 5). The cytoplasm of dead neurons showed the loss of Nissl granules (consisting of rough endoplasmic reticulum and polyribosomes), which is easily observed with the Klüver-Barrera (BK) stain. The perikaryon of degenerated or dead neurons stained turquoise blue (Figure 5 and Figure 6 black arrows). These findings correlate with HE-stained hippocampal tissue sections (data not shown). The neuropil (area between glial and neuronal cell bodies that is made up of dendrites, axons, glial cells and microvasculature) changed its tinctorial affinity. In photomicrographs of the superior lamina (SB-DG), hinge region (H-DG) and inferior lamina (IB-DG) of the dentate gyrus of the hippocampus, an increase in degenerated cells was not observed when compared with the CO group (Figure 6).

### 3.5. Permethrin Exposure Alters Mitochondrial Function

**Figure 7:**
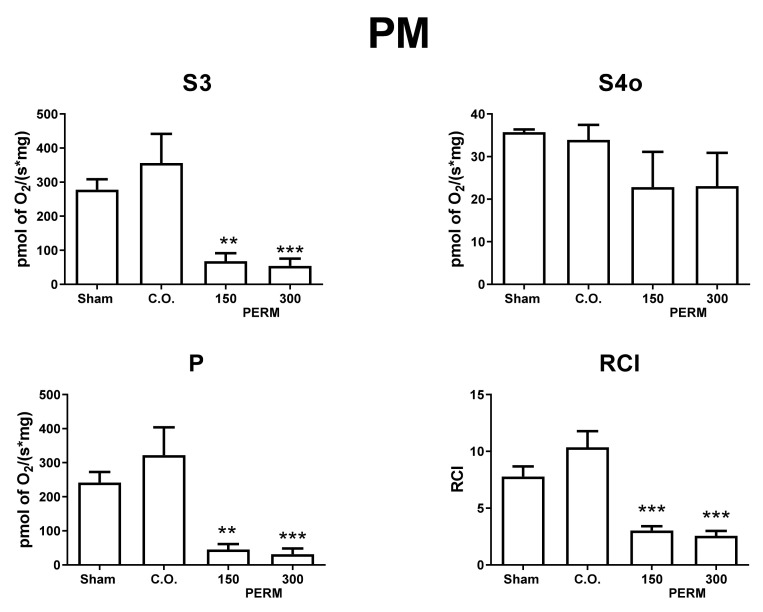
Respiratory parameters in CI-linked respiration. Mitochondrial respiratory parameters: state 3 (S3), state 4 induced by oligomycin (S4o), respiratory control index (RCI) and OXPHOS associated respiration (P) using malate-pyruvate (PM) as substrates. Data are mean ± SE, *n* = 6. ** *p* < 0.01 vs. sham, *** *p* < 0.001 vs. sham.

**Figure 8:**
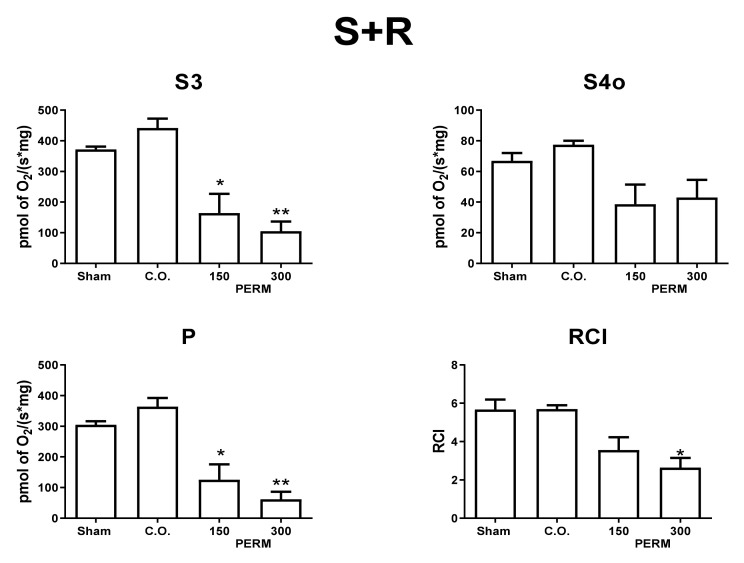
Respiratory parameters in CII-linked respiration. Mitochondrial respiratory parameters: state 3 (S3), state 4 induced by oligomycin (S4o), respiratory control index (RCI) and OXPHOS associated respiration (P) using succinate plus rotenone (S + R) as substrates. Data are mean ± SE, *n* = 5–6. * *p* < 0.05 vs. sham group, ** *p* < 0.01 vs. sham group.

To explore if hippocampal damage due to PERM is related to alterations in mitochondrial bioenergetics, we evaluated hippocampal mitochondrial respiratory parameters in CI- and CII-linked respiration (Figure 7 and Figure 8). PERM induced mitochondrial uncoupling and reduction of oxidative phosphorylation (OXPHOS) determined by *p* values. PERM significantly decreased the S3 and P respiratory parameters in CI and CII-linked respiration, starting at the lower dose. However, only the higher dose significantly reduced the RCI index of CII-linked respiration. Furthermore, the reduction in respiratory parameters was notably higher for CI-linked respiration.

These bioenergetics alterations suggest that PERM induced mitochondrial decoupling and a reduction of OXPHOS capacity, independently of the substrate type. Mitochondrial alterations could be related to a reduction in ATP synthase activity; however, taking into account the stronger effect in the respiration feed by PM, the results suggested that mitochondrial CI could be more affected. However, more experiments are needed to clarify this.

## 4. Discussion

Permethrin (PERM) is a synthetic pyrethroid extensively used to control agricultural pests and disease vectors. Originally it was considered safe for humans and other animals due to its relatively low environmental toxicity in mammals. However, its neurotoxicity at high doses was soon described, and more recently, several side effects at chronic low doses have been reported [10]. The main route for human exposure is through contaminated food ingestion such as fruits, vegetables, or milk where it can accumulate.

The neurotoxicity of PERM at high doses has been widely studied [16,17,18,20,21,22,23,24,25]. It was reported to modify sodium channels in insects and mammals, leading to prolonged depolarization and repetitive discharges in presynaptic nerve fibers after a single stimulus [14,15]. This action is associated with tremor, hyperactivity, ataxia, convulsions, and in some cases, paralysis. PERM can also inhibit neuronal activity in hippocampal glutamatergic networks in a potent and concentration-dependent manner. PERM increases the risk of neuronal deficit and the development of neurodegeneration [43,44]. The neurodegenerative mechanisms associated with PERM have been studied. Among them, a detriment to the redox system is one of the most reported. Oxidative stress is a major determinant of neurodegeneration; a pro-oxidant state can trigger posttranscriptional modifications in the electron transport system. This increases reactive oxygen species (ROS) production and inhibits mitochondrial complex I. Thus, the deficit of antioxidant enzymes in different organs leads to protein, lipid and DNA damage [25,28,31]. However, there is little information about PERM neurotoxicity after exposure to low doses and for short periods. In this study, the effect of sublethal doses (1/10 and 1/30 of the LD50) and subacute PERM treatment was evaluated to identify their impact on the redox, bioenergetic and immune systems. Sub-chronic PERM exposure at both doses increased lipoperoxidation and carbonyl protein (PC) content, hallmarks of oxidative stress induced by the pesticide, in the central nervous system. Previous studies have shown reactive oxygen species generation, such as superoxide anion (O_2_^●^^-^) hydrogen peroxide (H_2_O_2_), and nitric oxide (NO^●^) [45,46,47,48] in the brain due to PERM administration. In this regard, Falcioni and colleagues (2010) showed that sub-mitochondrial fractions of the striatum incubated with PERM showed decreased O_2_^●-^ levels [49]. On the other hand, carbonyl groups (aldehydes and ketones) are produced on protein side chains (especially in Pro, Arg, Lys and Thr residues) when they are oxidized [50,51,52]. Alternatively, PC can result from an indirect mechanism that involves hydroxyl radical-mediated lipid oxidation. Polyunsaturated acyl chains of phospholipids or polyunsaturated fatty acids, such as arachidonic acid and linoleic acid, are highly susceptible to peroxidation and breakdown via non-enzymatic Hock cleavage, forming a variety of lipid-derived aldehydes and ketones [52]. Our results suggest that sub-chronic PERM exposure at sublethal doses induces oxidative stress in the rat brain even when using 1/10 and 1/30 of the reported LD50.

Enzymatic antioxidant mechanisms in the brain are important in maintaining the balance between pro-oxidant and antioxidant agents and to reduce damage due to oxidative stress. The antioxidant enzymes superoxide dismutase, catalase and glutathione peroxidase are the first to act in the presence of ROS. They catalyze the breakdown of the dismutase superoxide radical, hydrogen peroxides and hydroperoxides into harmless molecules (H_2_O_2_/alcohol and O_2_^●-^) [53]. The enzymatic activity of SOD, CAT, GPx, GR and glutathione transferase (GST) were evaluated in the brain after sub-chronic and sublethal PERM exposure. After exposure, the activity of antioxidant enzymes increased, even with the lower dose used, in different regions of the nervous system; the highest increments were observed in SOD activity in all areas analyzed. Interestingly, the cerebellum showed major increases in the five enzymes studied.

Several studies have reported changes in the activity of SOD and GPx as well as a decay in GSH levels in the brain and peripherical blood after exposure to high doses of PERM [49,54,55]. Omotoso and co-workers explored SOD, CAT and GPx activities after PERM exposure in the prefrontal cortex, hippocampus and cerebellum correlated with lipoperoxidation using doses of 500 and 1000 mg/kg [56]. They found a general decrease in enzyme activity and a rise in lipoperoxidation levels. In our study, most of the antioxidant enzymes increased their activity in the four brain regions explored. However, when using 300 mg/kg, some enzymes reduced their activity or did not show changes, especially in the prefrontal cortex (Table 3). Moreover, macromolecular markers of oxidative stress damage such as lipoperoxidation increased in all brain areas and mainly in the hemispheres and medulla, while protein carbonylation showed a major increase in the prefrontal cortex. Considering all the data, PERM exposure could impair the enzymatic antioxidant system in a dose-dependent manner. Low doses may increase the activity of antioxidant enzymes, while high doses inhibit them. This change in the redox state can be considered to cause neuroinflammation.

On the other hand, lipoperoxidation and protein carbonylation have been linked to cell death. Our results showed that the hemisphere containing the cerebral cortex (parietal, entorhinal and occipital) and internal structures such as the hypothalamus and hippocampus is highly susceptible to oxidative damage. Similarly, the prefrontal cortex is also vulnerable to oxidative stress that could be generated by low PERM doses.

PERM increased ROS levels, which can trigger a cascade of molecular and transcriptional events. These include the production of proinflammatory cytokines from local microglia and infiltrative neutrophils, monocytes and lymphocytes [57,58,59]. Microglia are the main source of several cytokines, including interleukin-1β (IL-1β), tumor necrosis factor alpha (TNF-α) and interleukin-6 (IL-6) [60]. This is the first report of inflammatory markers (IL-1β, IL-6 and TNF-α gene expression) in various regions of the brain (cerebellum, medulla, cerebral cortex and hemispheres) after acute exposure to low PERM concentrations (150 or 300 mg/kg). Our results showed a significant increase in the expression of the three cytokines, mainly with the highest dose used. These pro-inflammatory cytokines participate in different processes such as neurogenesis, neurotransmission, cell proliferation and neuronal excitability [61,62,63,64].

Exposure to degenerated neurons, oxidized proteins, glycated products or lipid peroxidation induces a proinflammatory state in the adult brain. It has been demonstrated that high ROS levels increase IL-1β levels in hippocampal cultures, while antioxidant administration decreases their expression [65,66]. In turn, IL-1β significantly decreases cell proliferation in the hippocampus [67]. Additionally, PERM exposure induces a pro-inflammatory state that includes IL-6 production [68]. In the brain, IL-6 is produced in activated microglial cells and reduces proliferation, differentiation and survival of neuronal precursors in the dentate gyrus [69]. It also alters synaptic protein levels in the hippocampus [70]. Finally, results from studies in animal models showed that high levels of IL-6 can damage cognitive functions such as memory and learning [71].

TNF-α has been reported to have pro-and anti-neurogenic properties, depending on the concentration, experimental model and cell-derived regions [72,73,74]. In this sense, our results showed increased TNF-α mRNA expression at 300 mg/kg in the four brain regions evaluated, but no changes were detected at the 150 mg/kg concentration. In addition, it has been shown that PERM may directly activate microglial cells through its interaction with voltage-gated sodium channels (VGSC). This may contribute to excessive accumulation of intracellular Na^+^ that depolarizes the cells to release TNF-α [75]. Interestingly, this pro-inflammatory cytokine can alter the balance of excitatory to inhibitory neurotransmission, inducing a higher synaptic excitatory/inhibitory ratio [76]. Additionally, acute low doses of PERM induce neuronal degeneration in the brain [25] and increase spontaneous glutamate release from hippocampal neurons [77].

The most important increases in the analyzed cytokines were registered in cerebellum and medulla, suggesting a dysfunction of the normal physiology of these areas that may be related to ROS generated by low PERM exposure. Baek and co-workers concluded that the vulnerability to oxidative stress in the brain is region-specific and dependent on the local iron-catalyzed Fenton reaction or the Haber–Weiss reaction [78]. Other authors report that brain areas such as the cortex, hypothalamus, hippocampus and striatum are more susceptible to oxidative damage in comparison to the cerebellum. In this study, the qualitative histological analysis showed more damage in the hippocampus due to the administration of both doses of permethrin. Some of these changes, including the death or decrease in the number of neurons, were observed mainly in the dentate gyrus area. Abdel-Rahman and co-workers reported neuronal death induced by pesticides in a model of Gulf-War syndrome [79]. Parihar associated hippocampal pathology with decreased neurogenesis, loss of principal neurons, and inflammation as well as a dysfunction of mood and cognition in a model of Gulf-War illness using rats [28]. Our data showed that PERM exposure can compromise hippocampal structural integrity, which in turn could affect vital neurological functions of this brain region, causing alterations such as motor deficits or dysfunctions in learning and memory, as has been described by Omotoso and co-workers (2020) [56] and Abdel-Rahman and co-workers (2001) [79]. However, a more detailed study of the changes induced by PERM exposure is needed to better understand the effects of this pyrethroid on brain integrity.

Some studies report a relationship between oxidative stress and mitochondrial abnormalities in the brain. Moreover, it has been proposed that pyrethroids trigger an impairment of mitochondrial function, increasing ROS production. Similarly, mitochondria could be vulnerable to the deleterious effects of Ca_2_^+^ ions. The evaluation of mitochondrial bioenergetics parameters in the brain showed that PERM strongly decouples oxidative phosphorylation with the higher dose. This impairment may be associated with a reduction in the capacity for ATP synthesis, since there was an important decrease in *p* values and RCI independently of the substrate used (Figure 7 and Figure 8c,d). Furthermore, several models have demonstrated that an increase in oxidative stress can decrease the activity of ATP synthase and mitochondrial complexes [38,80]. Additionally, mitochondrial bioenergetic alterations can favor other mechanisms such as inflammation, fibrosis, and cell death and are involved in neurodegenerative disorders such as cancer, pulmonary diseases, diabetes, and cardiovascular diseases [81,82].

Complex I of the electron transport chain may be the most sensitive to PERM, and with both concentrations the same effect was observed. Our results agree with Gassner and coworkers [83], who reported a high susceptibility of complex I to the deleterious effects of permethrin in the rat liver. However, complex I was not the only affected complex, since state 3 was affected by PERM exposure even in the presence of rotenone. This suggests that PERM impairs other respiratory complexes.

Proton leakage was also analyzed by measuring O_2_ consumption under non-phosphorylating conditions (state 4o). The results did not show a statistically significant effect, but there was a tendency towards reduced oxygen consumption in hippocampal mitochondria isolated from animals treated with PERM. Oxidative phosphorylation produces superoxide and is the main source of ROS. Mitochondrial superoxide production is closely dependent on Δp, and Brand proposed that proton leakage could minimize oxidative damage by modulating superoxide production, where oxidative stress is considered a detrimental condition for normal brain functioning. Specifically, ROS increase the susceptibility to neuronal damage, and the variability in the data for state 4o could be related to differences in cell damage observed between the different hippocampal areas [84].

## 5. Conclusions

The results obtained in this study suggest that subacute PERM exposure at low doses increases ROS production, induces an imbalance in the enzymatic antioxidant system and increases the gene expression of pro-inflammatory interleukins in different brain areas. These changes could lead to cell damage mediated by mitochondrial functional impairment. These results allowed us to better understand the complex mechanism involved in the toxic effects induced by PERM. Subsequent experiments will explore minimal doses and determine the timing of the changes that we described in this study.

## Figures and Tables

**Table 1:** Oligonucleotides for gene expression assessment. From left to right: gene name, gene bank oligonucleotide (pb), TM (°C), primer sequence, forward and reverse.

Gene Name	GenBank Accession No.	Oligonucleotide (pb)	Tm (°C)	Forward	Reverse
IL-1β	NM_031512.2	228	60	GATGTTCCCATTAGACAGCTGCAC	GTCTTTCATCACACAGGACAGG
IL-6	NM_012589.1	221	54	TGGAGTTCCGTTTCTACCTGGAGT	TGGATGGTCTTGGTCCTTAGCCA
TNF-α	HQ201305.1	152	60	CTGGCCAATGGCATGGATCTCAAA	TGGTATGAAATGGCAAACCGGCTG
beta-actin	AF541940.1	217	60	CTGACAGGATGCAGAAGGAGAT	AGTAACAGTCCGCCTAGAAGCA

**Table 2:** Effect of permethrin exposure on lipoperoxidation of brain areas. MDA (nmoles /mL/mg of protein) levels in the different brain regions (cerebellum, cerebral cortex, cerebral hemispheres and medulla) of animals treated with permethrin (PERM 150 and 300 mg/kg) are shown as percentages (mean ± SD) of the levels detected in the vehicle and sham groups.

Groups	Cerebellum	Cerebral Cortex	Cerebral Hemispheres	Medulla
Sham	60.90 ± 1.89	69.53 ± 5.02	50.3 ± 3.28	40.63 ± 3.81
Corn oil (CO)	69.83 ± 5.11 ^ns^	72.53 ± 4.72 ^ns^	51.24 ± 4.7 ^ns^	48.34 ± 3.76 ^ns^
PERM 150	81.25 ± 5.43 **	85.45 ± 6.56 *	85.45 ± 6.56 **	64.90 ± 7.77 **
PERM 300	87.80 ± 4.88 **	97.77 ± 8.49 **	97.77 ± 8.49 ***	69.95 ± 4.06 ***

Effect of PERM on the cerebellum, prefrontal cortex, cerebral hemispheres and medulla among all groups. Without statistically different is showed as ns. It was statistically different between conditions: cerebellum. F(3,8) = 12.76, * *p* < 0.002; vs. sham; prefrontal cortex F(3,8) = 19.16, * *p* < 0.0005 vs. sham and ** *p* < 0.002 vs. sham; cerebral hemispheres F(3,8) = 12.76, ** *p* < 0.002 vs. sham and *** *p* < 0.001 vs. sham and medulla F(3,8) = 18.32, ** *p* < 0.0005 vs. sham and *** *p* < 0.0006 vs. sham. Each quantification was performed in triplicate using samples from six rats, and the values represent the mean ± SD. The differences were analyzed using ANOVAs followed by Bonferroni tests.

**Table 3:** Effect of permethrin (150 and 300 mg/kg body weight/day) on the activity of antioxidant enzymes in different brain tissues.

Treatment	GPx (U/mg/Prot)	GR (U/mg/Prot)	CAT (U/mg/Prot)	SOD (U/mg/Prot)	GST (µmol/CDNBmin/Prot)
Cerebellum					
Sham	0.019 ± 0.001	0.013 ± 0.001	8.90 ± 0.36	2.11 ± 0.171	0.036 ± 0.006
Corn oil	0.020 ± 0.002 ^ns^	0.014 ± 0.002 ^ns^	11.24 ± 0.81 ^ns^	3.06 ± 0.226 *	0.039 ± 0.016 ^ns^
PERM 150 mg/kg	0.026 ± 0.003 *	0.019 ± 0.003 *	13.96 ± 0.74 *	3.88 ± 0.594 **	0.054 ± 0.005 ^ns^
PERM 300 mg/kg	0.029 ± 0.004 **	0.021 ± 0.002 **	15.48 ± 1.05 **	3.92 ± 0.367 ***	0.068 ± 0.005 **
Prefrontal cortex					
Sham	0.012 ± 0.006	0.015± 0.006	13.50 ± 0.71	1.19 ± 0.052	0.021 ± 0.001
Corn oil	0.015 ± 0.001 ^ns^	0.010 ± 0.001 ^ns^	14.03 ± 0.88 ^ns^	1.23 ± 0.14 ^ns^	0.027 ± 0.003 ^ns^
PERM 150 mg/kg	0.017 ± 0.009 **	0.015 ± 0.001 ^ns^	17.98 ± 1.30 **	1.86 ± 0.14 ***	0.032 ± 0.004 **
PERM 300 mg/kg	0.016 ± 0.001 ^ns^	0.014 ± 0.002 ^ns^	16.66 ± 1.34 *	1.73 ± 0.14 **	0.033 ± 0.004 **
Cerebral hemispheres					
Sham	0.012 ± 0.001	0.010 ± 0.000	7.24 ± 0.06	0.86 ± 0.005	0.020 ± 0.003
Corn oil	0.013 ± 0.001 ^ns^	0.010 ± 0.001 ^ns^	7.75 ± 0.65 ^ns^	0.97 ± 0.144 *	0.022 ± 0.002 ^ns^
PERM 150 mg/kg	0.015 ± 0.002 *	0.010 ± 0.001 ^ns^	10.80 ± 0.84 *	1.09 ± 0.045 **	0.029 ± 0.001 ^ns^
PERM 300 mg/kg	0.016 ± 0.001 **	0.010 ± 0.001 ^ns^	10.76 ± 0.73 *	1.12 ± 0.0640 *	0.029 ± 0.002 ^ns^
Medulla					
Sham	0.022 ± 0.000	0.062 ± 0.003	9.97 ± 0.75	3.78 ± 0.66	0.021 ± 0.001
Corn oil	0.021 ± 0.001 ^ns^	0.070 ± 0.001 ^ns^	11.07 ± 0.90 ^ns^	4.98 ± 0.98 ^ns^	0.018 ± 0.004 ^ns^
PERM 150 mg/kg	0.025 ± 0.002 ^ns^	0.076 ± 0.00 *	12.39 ± 0.91 *	6.72 ± 1.4 *	0.020 ± 0.003 ^ns^
PER 300 mg/kg	0.025 ± 0.002 ^ns^	0.080 ± 0.001 **	12.88 ± 0.99 **	7.7 ± 1.00 **	0.018 ± 0.003 ^ns^

The results were analyzed with ANOVAs; Bonferroni’s multiple comparison test was used to compare the outcomes between the experimental and Sham groups. Mean ± standard deviations (*n* = 6 per group). Without statistically different is showed as ns. *** *p* < 0.0001 vs. sham group; ** *p* < 0.001 vs. sham group, * *p* < 0.01 vs. sham group; CO: Corn oil, CAT: Catalase, GPx: Glutathione peroxidase, GR: Glutathione reductase, SOD: Superoxide dismutase (all in U/mg protein), GST: Glutathione-S-transferase (Umol/CDNB conjugate formed/min/mg protein) and PERM: Permethrin.

## Data Availability

Data is contained within the article.
